# A giant hiatal hernia with a congenital diaphragmatic hernia in a pediatric patient with arterial tortuosity syndrome: a case report

**DOI:** 10.3389/fped.2026.1750672

**Published:** 2026-02-10

**Authors:** Fatimah Jafer Almabyouq, Zahrh Faisal Abualsaud, Riyadh Mohammed Ali Alabbas

**Affiliations:** 1 Department of Pediatric Surgery, Imam Abdulrahman Bin Faisal University, Dammam, Saudi Arabia; 2 Emergency Medicine Department, Dammam Medical Complex, Dammam, Saudi Arabia

**Keywords:** arterial tortuosity syndrome, congenital diaphragmatic hernia, giant hiatal hernia, GLUT10, SLC2A10 gene

## Abstract

Congenital diaphragmatic hernia (CDH) is a rare condition, with many affected patients remaining asymptomatic, while others may present with non-specific respiratory manifestations. The coexistence of a CDH and a giant hiatal hernia is particularly uncommon. We report the case of a pediatric patient presenting with both a giant hiatal hernia and a CDH, accompanied by distinctive facial features. The initial presentation included poor oral intake and abnormal chest sounds, as observed by the patient's mother. A diagnostic evaluation using chest radiography and computed tomography confirmed the presence of both hernias. Subsequent genetic testing identified arterial tortuosity syndrome. The patient underwent primary surgical repair of both defects, along with partial fundoplication. The procedure was uneventful, and postoperative monitoring was carried out closely. Postoperatively, the patient developed dumping syndrome, which was successfully managed with octreotide. The patient demonstrated excellent clinical outcomes during follow-up.

## Introduction

Congenital diaphragmatic hernia (CDH) and hiatal hernia are clinically significant disorders of the diaphragm encountered in the pediatric population, both involving abnormal communication between the thoracic and abdominal cavities.

CDH is a developmental anomaly resulting from failure of closure of the pleuroperitoneal folds, leading to a diaphragmatic defect through which abdominal viscera herniate into the thoracic cavity. This displacement interferes with normal pulmonary development, often resulting in pulmonary hypoplasia and persistent pulmonary hypertension after birth. Affected neonates typically present with severe respiratory distress and a scaphoid abdomen shortly after delivery.

Hiatal hernia in children is characterized by herniation of the stomach through the esophageal hiatus into the thoracic cavity and is frequently associated with gastroesophageal reflux disease. Clinical manifestations range from asymptomatic cases to reflux symptoms, feeding difficulties, and respiratory compromise. Diagnosis relies primarily on radiological imaging and, in select cases, endoscopic evaluation.

Despite advances in prenatal diagnosis and neonatal intensive care, CDH remains associated with substantial morbidity and mortality. Management requires stabilization followed by surgical repair once the infant is clinically optimized. In contrast, management of hiatal hernias may be either conservative or surgical, depending on symptom severity and associated complications.

## Case

A 3-month-old female infant, born at term via spontaneous vaginal delivery following an uneventful pregnancy, was referred to the pediatric surgery clinic with poor oral intake, a thin abdomen, and abnormal chest sounds noted by her mother. There was no history of choking episodes, vomiting, recurrent aspiration, respiratory infections, or fever.

The parents, who were first-degree cousins, reported a notable family history of congenital anomalies, including diaphragmatic hernias in three cousins who had undergone surgical repair with favorable outcomes and cardiac anomalies characterized by tortuous pulmonary arteries.

On physical examination, the patient appeared comfortable, alert, and well-perfused. She was pink and well-nourished, with distinctive facial features including micrognathia and a high-arched palate. Arachnodactyly was noted in both hands, along with hyperextensible skin.

The patient’s vital signs were within normal limits, and she weighed 4.6 kg and measured 64 cm in length. A chest examination revealed symmetrical expansion during inspiration without visible deformity; bowel sounds were audible over the left hemithorax. An abdominal examination demonstrated a scaphoid, soft, and lax abdomen without organomegaly. The hernial orifices were intact, and the external genitalia were normal.

**Figure 1:**
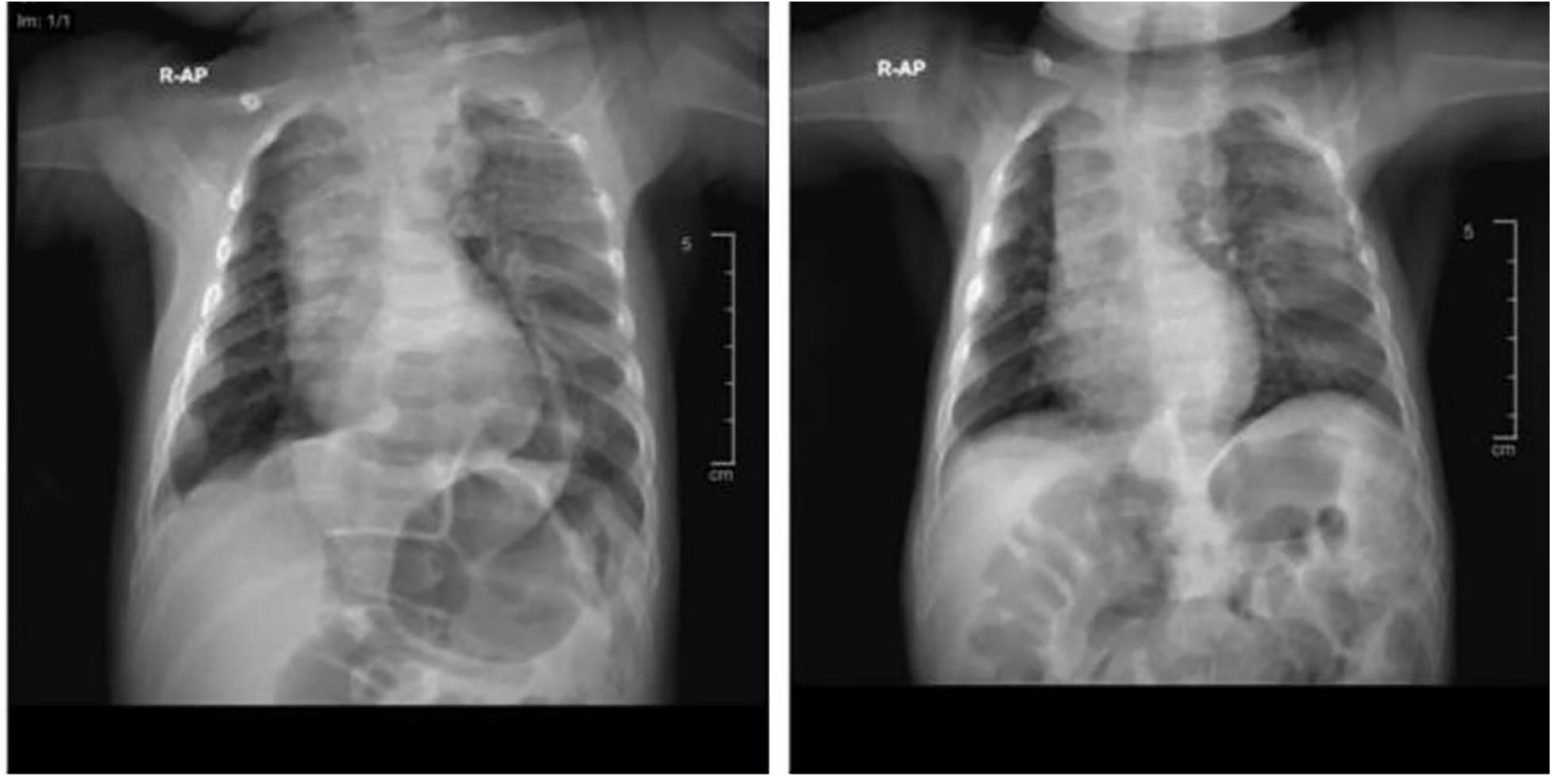
Anteroposterior chest X-rays of the patient **(A)** at the time of diagnosis and **(B)** 2 months postoperatively.

A chest X-ray (anteroposterior view) showed an air-filled opacity in the retrocardiac region and the left lower lung zone (Figure 1A).

**Figure 2:**
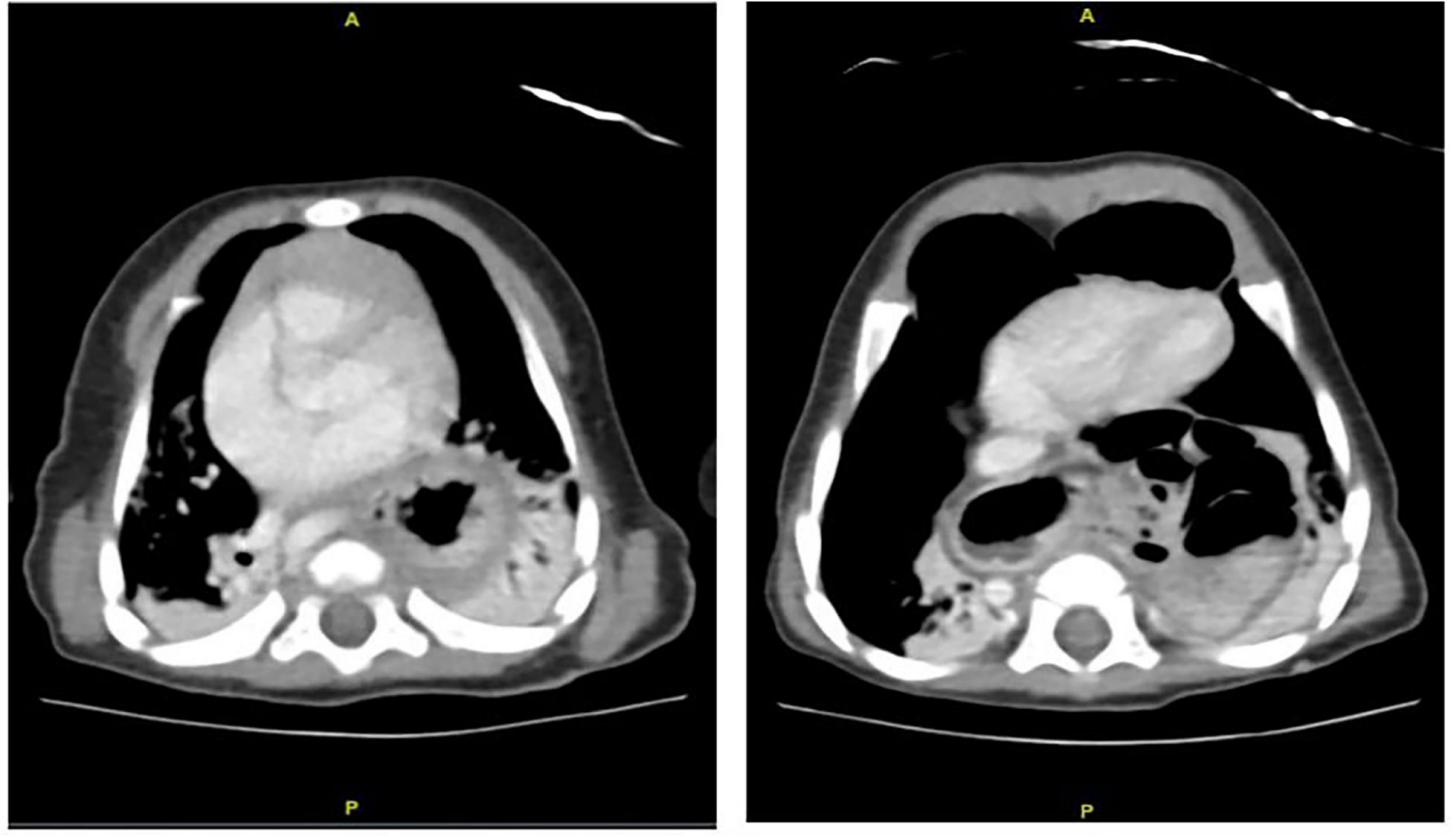
CT scan of the chest and abdomen of the patient shows a paraesophageal hernia with an approximately 3.5 cm defect, associated with superior herniation of the stomach and bowel loops. Another defect is noted at the diaphragm anterior to the heart, measuring approximately 3.22 cm, associated with herniation of the transverse colon into the anterior mediastinum, representing a Morgagni hernia. There is a bilateral consolidation and atelectasis within the lower lobes.

Further evaluation with contrast-enhanced CT of the chest and abdomen revealed a paraesophageal hernia with an approximately 3.5 cm defect, with herniation of the stomach and bowel loops into the mediastinum. An additional diaphragmatic defect consistent with a Morgagni hernia was identified, associated with herniation of the transverse colon into the anterior mediastinum. Elongation and tortuosity of the aorta and major abdominal and pelvic vessels were also observed, without evidence of aneurysmal dilatation (Figure 2).

**Figure 3:**
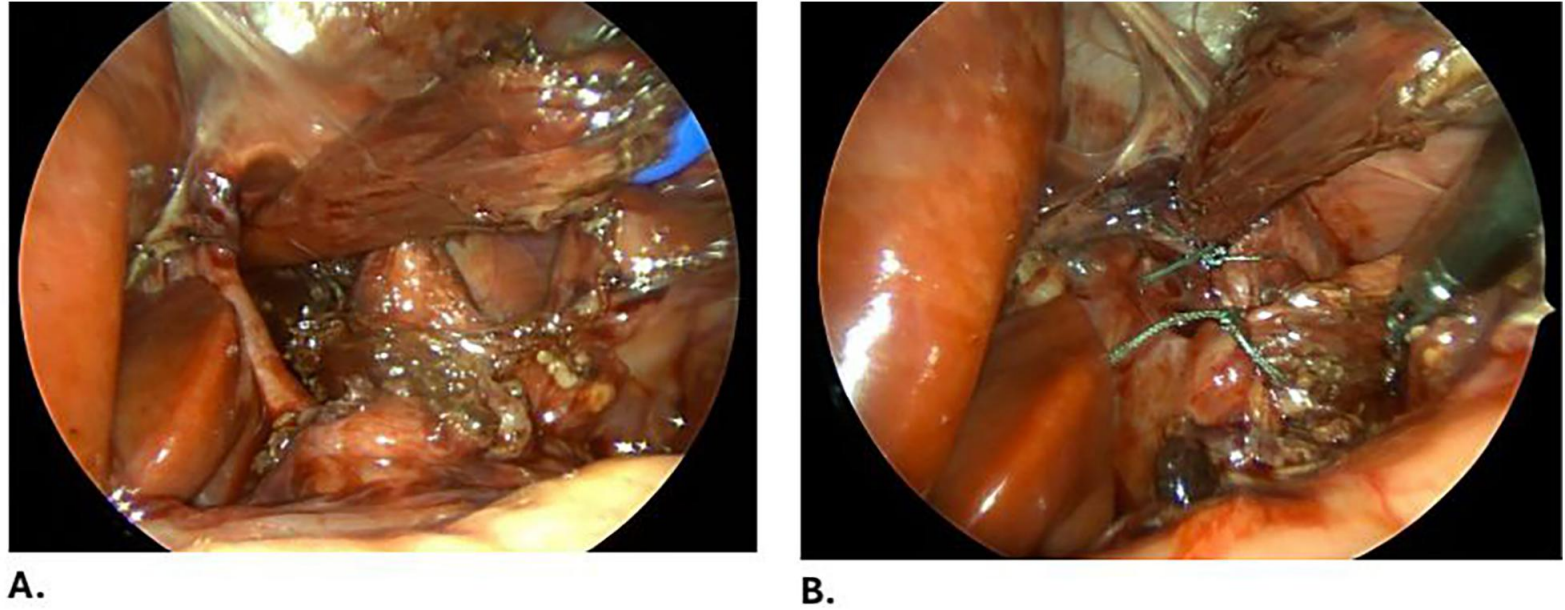
**(A)** A giant hiatal hernia defect, showing the crura. **(B)** The defect is closed using Ethibond [Ethibond Excel 2-0 TM, Ethicon LLC (Johnson & Johnson Medical GmbH), Norderstedt, Germany] and posterior Toupet fundoplication is performed.

**Figure 4:**
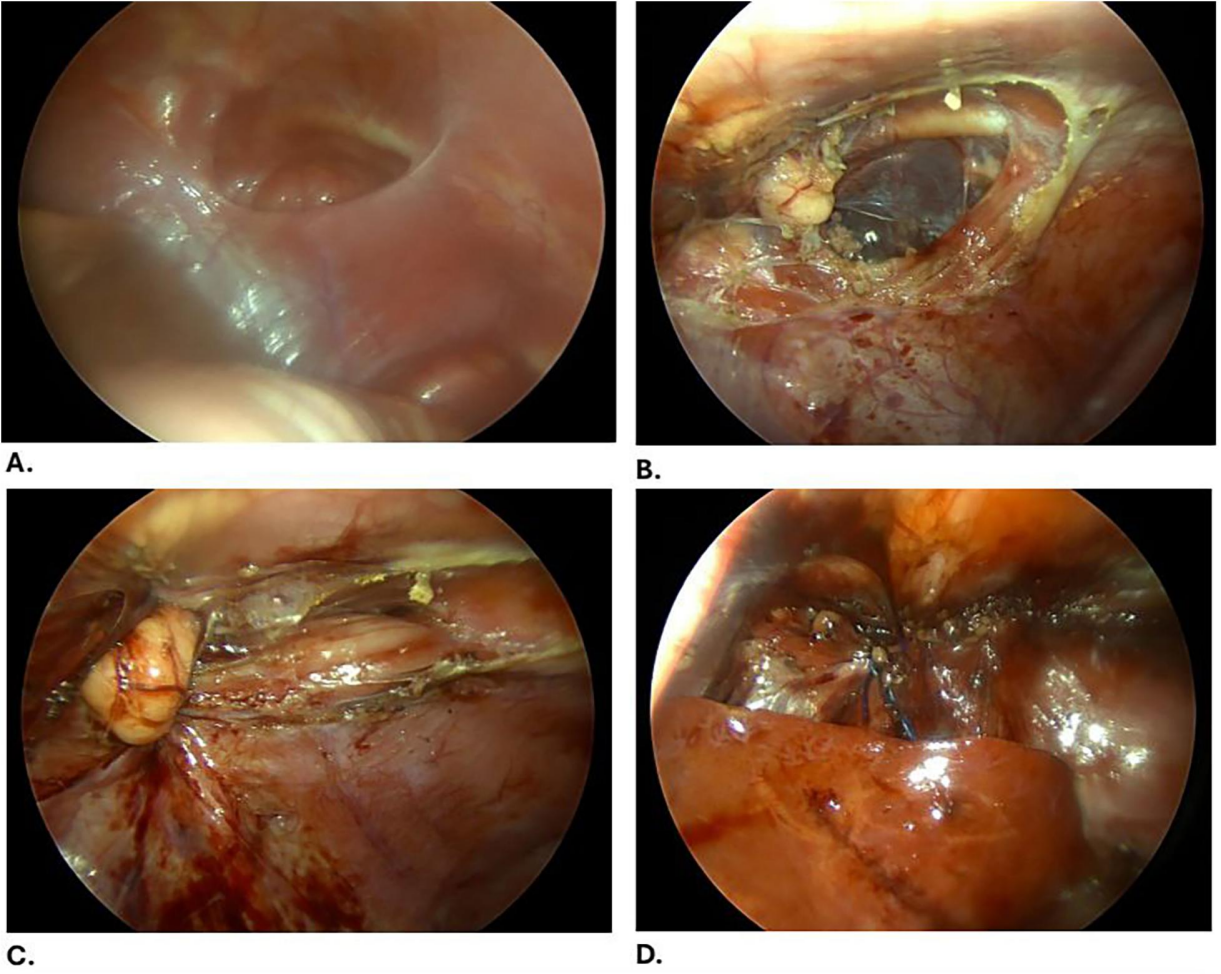
**(A)** A Morgagni hernia is evident with an empty hernia sac. **(B)** Undermining the surrounding edges of the defect. **(C)** Primary closure of the defect with needle-assisted technique using 1-0 Prolene sutures. **(D)** Reinforcement is performed using a V-Loc suture.

A diagnosis of a giant hiatal hernia and a congenital diaphragmatic hernia was established. An evaluation by a pediatric cardiologist, supported by transthorasic echocardiography, revealed tortuosity of the aortic arch, head and neck vessels, and pulmonary artery branches, without obstruction or aneurysmal changes.

Following cardiology clearance, the patient was electively admitted for surgical management. A diagnostic laparoscopy was performed using four 3-mm ports. Intraoperatively, a giant hiatal hernia was identified, with a hernia sac containing the stomach, portions of the transverse colon, small bowel loops, and the left lobe of the liver. A Morgagni hernia with an empty sac was also noted. The herniated contents were reduced, the hiatal hernia sac was dissected and excised, and the defect was repaired by approximation of the diaphragmatic crura using non-absorbable sutures (Figure 3). A partial posterior Toupet fundoplication was performed due to the tubular configuration of the stomach. The Morgagni defect was closed primarily with 1-0 Prolene sutures [Prolene 1-0 TM, Ethicon LLC (Johnson & Johnson Medical GmbH), Norderstedt, Germany] and reinforced with a V-Loc suture [V-Loc Covidien LP (Medtronic), Mansfield, MA] (Figure 4).

The patient tolerated the procedure well and was transferred to the pediatric intensive care unit for postoperative observation. On postoperative day 1, the patient developed atelectasis, which was managed conservatively with chest physiotherapy. The nasogastric tube was removed on postoperative day 3, and oral feeding was resumed. Recurrent episodes of hypoglycemia prompted a consultation with a pediatric endocrinologist. Modification of the feeding formula resulted in partial improvement, and octreotide therapy was initiated.

The patient was discharged in a stable condition on postoperative day 25, with scheduled follow-ups in the endocrinology and genetics clinics. Follow-up evaluations at 2 and 6 weeks demonstrated satisfactory recovery and appropriate weight gain, with no surgical complications. A chest X-ray obtained 2 months postoperatively confirmed radiological improvement (Figure 1B). At 9 months of age, the patient remained thin but exhibited a good appetite and was referred to a dietitian prior to discharge from surgical follow-up.

Genetic testing using whole exome sequencing (CentoXome test) identified a homozygous pathogenic variant in the *SLC2A10* gene, which encodes for the GLUT10 protein. This *SLC2A10* variant, c.243C>G p.(Ser81Arg), causes substitution of the amino acid serine (Ser) with arginine (Arg) at position 81 of the protein. This alteration occurs in exon 2 (of a total of five exons) in the gene, causing a loss-of-function mutation of the GLUT10 protein and confirming the diagnosis of autosomal recessive arterial tortuosity syndrome (ATS; PMID:16550171, 36578839, 29323665). Both parents were found to carry the same gene, and their cousins were undergoing genetic analysis.

## Discussion

CDH is a rare developmental anomaly that results from the pleuroperitoneal folds failing to close between the 4th and 10th weeks of gestation (1). Herniation of abdominal viscera into the thoracic cavity may occur through the diaphragmatic hiatus, through congenital defects such as the foramina of Bochdalek and Morgagni, or through acquired diaphragmatic disruption (1). Congenital diaphragmatic hernias classically occur through the Bochdalek and Morgagni defects (1).

The reported incidence of CDH ranges from approximately 1 in 2,000 to 1 in 5,000 live births, whereas hiatal hernias in the pediatric population are less common and are frequently identified incidentally during upper gastrointestinal imaging studies.

There is no specific presentation for a congenital diaphragmatic hernia. The majority of affected patients remain asymptomatic, while others may present with respiratory symptoms during childhood (2, 3). In addition, symptoms such as vomiting, recurrent chest infections, and reduced oral intake should raise clinical suspicion for CDH.

Laboratory investigations are typically within normal limits. Several radiological modalities are available for diagnosis, with anteroposterior and lateral chest radiographs serving as the most common initial investigations, followed by computed tomography, ultrasonography, and upper gastrointestinal contrast studies, all of which have demonstrated diagnostic utility (1, 4–6). Furthermore, CDH may be detected antenatally during routine anomaly scans performed between 20 and 24 weeks of gestation.

Surgical management involves anatomical repair of the diaphragmatic defect, which may be achieved either laparoscopically or via an open approach. A mesh may be necessary to close large diaphragmatic defects. In cases involving a hiatal hernia, fundoplication is essential to reduce the risk of postoperative gastroesophageal reflux. Postoperative intensive care monitoring is necessary to detect early complications, particularly pulmonary hypertension.

The coexistence of a Morgagni hernia and a hiatal hernia is uncommon, but has been described in the literature. At our institution, we previously reported on an incidental Morgagni hernia discovered during the laparoscopic repair of a hiatal hernia in an adult patient, with both defects repaired simultaneously via a minimally invasive approach (7).

In the present case, the patient presented at a relatively late stage with decreased oral intake and abnormal chest sounds. Physical examination revealed a scaphoid abdomen, skin hyperlaxity, and arachnodactyly. Computed tomography findings demonstrating arterial tortuosity, combined with a significant family history of CDH and congenital cardiac anomalies, prompted further investigation for an underlying genetic disorder. Subsequent genetic testing confirmed the diagnosis of ATS.

ATS is an extremely rare connective tissue disorder characterized by the elongation and tortuosity of medium- and large-sized arteries (8). The condition is caused by loss-of-function mutations in the *SLC2A10* gene, which encodes the GLUT10 glucose transporter (9). GLUT10 is highly expressed in lung alveoli and has been implicated in maintaining airway integrity, which may explain its frequent association with infant respiratory distress syndrome in affected patients (10). Diaphragmatic hernias and sliding hiatal hernias have been reported in up to 50% of individuals with ATS (11).

To our knowledge, this is the first reported case in English describing a patient with arterial tortuosity syndrome presenting with both a giant hiatal hernia and a congenital diaphragmatic hernia. Beyens et al. reported the presence of isolated inguinal hernias or the coexistence of either a hiatal hernia or CDH in patients with ATS, but not the simultaneous occurrence of both diaphragmatic defects (10). A detailed report described a newborn diagnosed with ATS who exhibited multiple vascular malformations, a hiatal hernia, and recurrent bilateral inguinal hernias requiring surgical repair; unfortunately, the patient later died from septic shock secondary to bowel obstruction (9).

Wahab et al. reported on a cohort of 32 patients from a large, interrelated family in Qatar, all of whom exhibited characteristic features, including skin hyperextensibility, hypotonia, joint hypermobility, and tortuous systemic arteries. An inguinal hernia was observed in 11 patients, while diaphragmatic and/or hiatal hernias were identified in seven patients. Parental consanguinity was present in all cases and was traced to a common ancestor within a large Bedouin tribe, with all affected individuals harboring *SLC2A10* mutations (12).

Similarly, Fadley et al. described a series of 12 patients from Saudi Arabia with severe arterial tortuosity and stenoses involving systemic, pulmonary, and coronary vessels. Although these patients shared phenotypic overlap with the present case, no hernias, respiratory complications, or hypotonia were reported (13).

## Conclusion

The simultaneous occurrence of a congenital diaphragmatic hernia and a giant hiatal hernia is rare and may be easily overlooked during childhood due to the absence of classic symptoms. A delayed diagnosis increases the risk of preventable complications. Early recognition, appropriate imaging, and timely surgical intervention are essential to optimize outcomes, particularly in patients with underlying genetic connective tissue disorders.

## Data Availability

The raw data supporting the conclusions of this article will be made available by the authors, without undue reservation.
